# Clinical Potential of Hypoxia Inducible Factors Prolyl Hydroxylase Inhibitors in Treating Nonanemic Diseases

**DOI:** 10.3389/fphar.2022.837249

**Published:** 2022-02-24

**Authors:** Mengqiu Miao, Mengqiu Wu, Yuting Li, Lingge Zhang, Qianqian Jin, Jiaojiao Fan, Xinyue Xu, Ran Gu, Haiping Hao, Aihua Zhang, Zhanjun Jia

**Affiliations:** ^1^ Department of Nephrology, Children’s Hospital of Nanjing Medical University, Nanjing, China; ^2^ Nanjing Key Laboratory of Pediatrics, Children’s Hospital of Nanjing Medical University, Nanjing, China; ^3^ Jiangsu Key Laboratory of Pediatrics, Nanjing Medical University, Nanjing, China; ^4^ School of Medicine, Southeast University, Nanjing, China; ^5^ State Key Laboratory of Natural Medicines, Key Laboratory of Drug Metabolism, China Pharmaceutical University, Nanjing, China

**Keywords:** hypoxia inducible factors (HIFs), HIF-PHDs inhibitors (HIF-PHIs), nonanemic diseases, roxadustat (FG-4592), daprodustat (GSK-1278863), vadadustat (AKB-6548), molidustat (BAY 85-3934), enarodustat (JTZ-951)

## Abstract

Hypoxia inducible factors (HIFs) and their regulatory hydroxylases the prolyl hydroxylase domain enzymes (PHDs) are the key mediators of the cellular response to hypoxia. HIFs are normally hydroxylated by PHDs and degraded, while under hypoxia, PHDs are suppressed, allowing HIF-α to accumulate and transactivate multiple target genes, including erythropoiesis, and genes participate in angiogenesis, iron metabolism, glycolysis, glucose transport, cell proliferation, survival, and so on. Aiming at stimulating HIFs, a group of small molecules antagonizing HIF-PHDs have been developed. Of these HIF-PHDs inhibitors (HIF-PHIs), roxadustat (FG-4592), daprodustat (GSK-1278863), vadadustat (AKB-6548), molidustat (BAY 85-3934) and enarodustat (JTZ-951) are approved for clinical usage or have progressed into clinical trials for chronic kidney disease (CKD) anemia treatment, based on their activation effect on erythropoiesis and iron metabolism. Since HIFs are involved in many physiological and pathological conditions, efforts have been made to extend the potential usage of HIF-PHIs beyond anemia. This paper reviewed the progress of preclinical and clinical research on clinically available HIF-PHIs in pathological conditions other than CKD anemia.

## 1 Introduction

Cells sense and adapt to hypoxia through the activity of hypoxia inducible factors (HIFs) and their regulatory hydroxylases, three HIF prolyl hydroxylase domain enzymes (PHD1-3) and an asparaginyl hydroxylase (factor-inhibiting HIF, or FIH). Under normal cellular oxygen concentrations, HIF-α is dual inhibited by PHDs and FIH. PHDs represses the abundance of HIF-α by hydroxylating HIF-α at highly conserved proline residues in the N- or C-terminal oxygen-dependent degradation domains, and the resulting hydroxylated proteins combine with the von Hippel-Lindau (VHL) E3 ubiquitin ligase complex and are ultimately degraded by the proteasome (Beck et al., 2017). Meanwhile FIH hampers the transcriptional activity of HIF-α by blocking the interaction between HIF-α and transcriptional co-activator histone acetyltransferase p300/CREB-binding protein, via hydroxylating HIF-α at asparagine803 located in the C-terminal transactivation domain (Wong et al., 2013; Strowitzki et al., 2019). Instead, under hypoxic conditions, PHDs and FIH are suppressed, allowing HIF-α to accumulate in the nucleus and dimerize with HIF-β, forming transcriptionally active HIFs and transactivating hundreds of target genes to restore tissue homeostasis (Schofield and Ratcliffe, 2004).

Hypoxia stimulates HIFs and then interacts with the upstream binding sites (called HIF regulatory elements) of various target genes to activate transcription. Specifically, HIF-1 appears to be expressed in nearly all cell types and masters the expression of genes involved in glycolytic metabolism (Kim et al., 2006; Semenza, 2006; Semenza, 2017) and mitochondrial metabolism (Suda et al., 2011), thus promoting anaerobic glycolysis and preventing pyruvate from becoming decarboxylated and entering the Kreb’s cycle (Mazure and Pouyssegur, 2010). Additionally, HIF-1 controls mitochondrial health through modulation of critical genes involved in the mitophagic pathway (BCL2/adenovirus E1B 19 kDa protein-interacting protein 3 (BNIP3) and BNIP3 L) (Semenza, 2012). Conversely, HIF-2α is expressed in a more cell-restricted manner and preferentially promotes the expression of genes dictating erythropoiesis (EPO) and iron homeostasis (Rankin et al., 2007; Kapitsinou et al., 2010; Kobayashi et al., 2016). Both HIF-1α and HIF-2α regulate the expression of vascular endothelial growth factor (VEGF), the main regulator of angiogenesis, and some other target genes (Keith et al., 2011).

Hypoxia is involved in the pathology of various diseases ranging from ischemia injuries to infectious diseases, and pharmacological stimulation of HIF activity represents an effective therapeutic approach in a portion of these diseases. By inhibiting PHDs, HIF prolyl hydroxylase inhibitors (HIF-PHIs) stabilize HIFs, allowing HIFs to act on downstream target genes. HIF-PHIs stimulated HIFs, allowing HIF-2 to act on EPO-producing cells in the kidney and liver to promote endogenous EPO production and subsequent hematopoiesis. In addition, stimulated HIFs also regulate iron-related protein expression involved in iron metabolism and utilization (Peyssonnaux et al., 2007; Mastrogiannaki et al., 2012; Gupta and Wish, 2017; Haase, 2017). Of these HIF-PHIs, roxadustat (FG-4592) was first approved for the clinical treatment of anemia in chronic kidney disease (CKD) patients in China and other Asia-Pacific countries (Yap et al., 2021). Afterwards, other HIF-PHIs, such as daprodustat (GSK-1278863), vadadustat (AKB-6548), molidustat (BAY 85-3934) and enarodustat (JTZ-951), have passed or are going through different clinical trials for ulteriorly usage in the future. While the effectiveness and safety of JNJ-42905343 (Janssen Pharmaceuticals, Raritan (HQ), NJ. United States), DS-1093 (Daiichi Sankyo, Inc., Chuo City, Tokyo, Japan, no longer investigated for anemia) and Zyan1 (Cadila Healthcare Ltd., Ahmedabad, Gujarat, India) are still in preclinical, phase 1 or 2 clinical trials for renal anemia or are being evaluated for other indications. Other HIF-PHIs such as dimethyloxalylglycine (Trichonas et al., 2013; Xie et al., 2014), L-mimosine (L-mim) (Trimmel et al., 2015; Janjic et al., 2019), MK8617 (Debenham et al., 2016; Li et al., 2019) and an FIH selective inhibitor N-oxalyl-D-phenylalanine (Meng et al., 2018) are still in preclinical research. Since the action and mechanism of HIF-PHIs in CKD anemia are well summarized in reviews published before (Forristal and Levesque, 2014; Gupta and Wish, 2017; Haase, 2017; Locatelli et al., 2017; Del Vecchio and Locatelli, 2018; Sanghani and Haase, 2019), this article focuses on milestones in the development of these clinical available HIF-PHIs and their potential in treating non-anemic diseases.

## 2 Clinical Available HIF-PHDs Inhibitors

Firsly, the clinical research stage, molecular characteristics, mechanisms of action, potency against different PHDs, and limitations of five clinical available HIF-PHIs are summarized below and in Table 1.

**TABLE 1:** Molecular character, mechanisms of action, development status and main limitation of five clinical available HIF-PHIs.

Product	Enzyme inhibition	Development status	Diseases	Results	Limitation
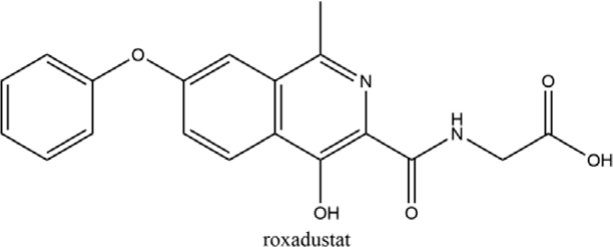	HIF-PHD 1,2,3	Phase 3 or completed	• Anemia in DD- or NDD-CKD patients (AstraZeneca; The Third Affiliated Hospital of Chongqing Medical University)	• Increase Hb level	• Analyses for thromboembolic adverse events displayed
			• Myelodysplastic Syndrome (FibroGen Inc.)	• Decrease hepcidin levels	• Further assessment of efficacy and safety in need
				• Decrease total cholesterol and triglycerides levels	
				• Inflammation shows no impact on therapeutic effects	
				• Reducing transfusions in low-risk myelodysplastic syndrome	
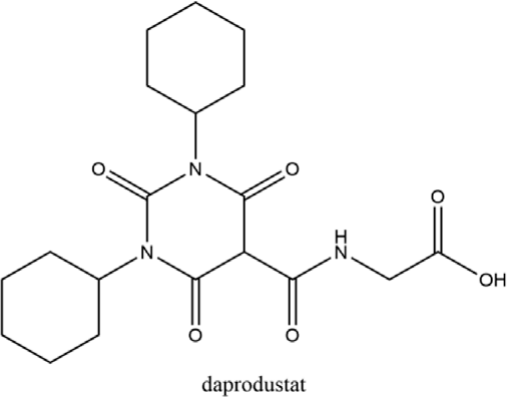	HIF-PHD 1,3	Phase 3	Anemia in DD- or NDD-CKD patients	• Increase Hb levels	• Hyperkalemia and increased BP observed
			(GlaxoSmithKline Research & Development Ltd.)	• Decrease hepcidin levels	• Tumor effect in NDD patients observed
					• Further assessment of efficacy and safety in need
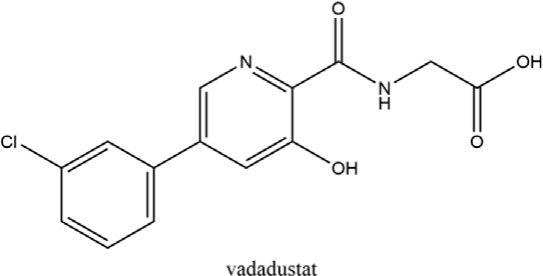	Not specific	Phase 2/3	Anemia in DD- or NDD-CKD patients	• Increase Hb levels	• Effects on cholesterol and inflammation not reported
			(Akebia Therapeutics)	• Decrease hepcidin levels	• Gastrointestinal reactions, hypertension and hyperkalemia reported more frequently
					• Further assessment of efficacy and safety in need
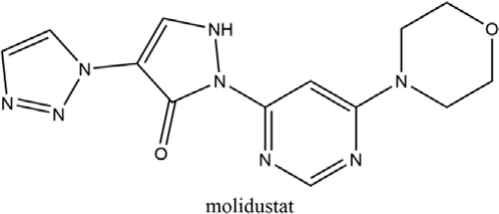	HIF-PHD 2	Phase 2/3	Anemia in DD- or NDD-CKD patients	• Increase Hb levels	• Effects on cholesterol and inflammation not reported
			(Bayer)	• Decrease hepcidin levels	• Further assessment of efficacy and safety in need
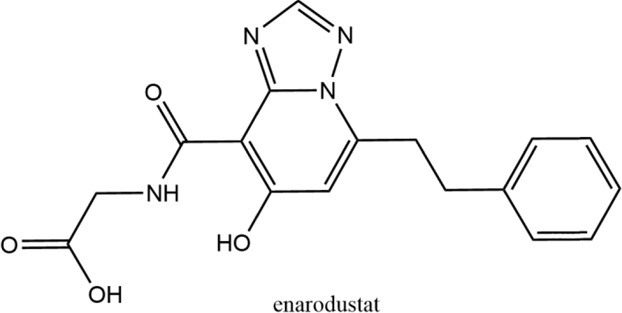	Not specific	Phase 3	Anemia in DD- or NDD-CKD patients	• Increase Hb levels	• Effects on iron metabolism and inflammation not reported
			(Japan Tobacco Inc.)		• Further assessment of efficacy and safety in need

### 2.1 Roxadustat (FG-4592)

Roxadustat, an orally administered HIF-PHI from FibroGen (San Francisco, United States), Astellas (Northbrook, Illinois, United States), and AstraZeneca (Wilmington, DE, United States), targets all three PHDs to a similar extent and is usually dosed three times weekly in clinical use (Yeh et al., 2017). Completed over thirty phase 3 studies (Dhillon, 2019), roxadustat has become the first-in-class compound that has achieved formal marketing authorization by the National Medical Products Administration for the treatment of anemia in patients with later-stage CKD who are dialysis- or not dialysis-dependent in China. Studies demonstrated that roxadustat showed a dose-dependent effect on erythropoiesis while maintaining plasma erythropoietin levels within or near the normal physiologic range, including in the presence of inflammation and iron metabolism (Besarab et al., 2015; Provenzano et al., 2016; Liu J. et al., 2020).

Numerous studies (Provenzano et al., 2016; Chen et al., 2019a; Chen et al., 2019b; Liu J. et al., 2020) have demonstrated that both in the dialysis-dependent (DD)-CKD patients and in the non-dialysis-dependent (NDD)-CKD patients, roxadustat showed a significant effect on erythropoiesis while maintaining plasma erythropoietin levels within or near the normal physiologic range. Several publications indicated that the effective increase in Hemoglobin (Hb) may be associated with the regulation of iron metabolism, which presents the reduction in plasma hepcidin levels along with decreases in plasma ferritin and an increase in total iron binding capacity (Chen et al., 2019a; Chen et al., 2019b; Akizawa et al., 2020a; Akizawa et al., 2020c; Shutov et al., 2021). However, analyses for thromboembolic adverse events in roxadustat application of NDD and DD population displayed a fairly strong association between the application dosage and thromboembolic events in roxadustat-treated subjects (Food and Drug Adiministration, 2021a). Some committee members of FDA thus questioned its efficacy and safety and urged more clinical evidence prior to approval (Food and Drug Administration, 2021b). On the contrary, roxadustat showed no effects on platelet production, activation, and thrombosis formation in healthy and 5/6 nephrectomized mice (Zhao et al., 2021). Therefore, more experiments are awaited with interest.

In addition to CKD anemia, a phase 3 study was carried out to assess the efficacy and safety of roxadustat in patients with low-risk myelodysplastic syndrome recently obtained preliminary satisfactory results in reducing transfusions (Henry et al., 2021). More immature but promising therapeutic strategies are under consideration and tested in preclinical studies, which will be discussed in detail hereinafter.

### 2.2 Daprodustat (GSK-1278863)

Daprodustat, a once daily oral HIF-PHI from GlaxoSmithKline, Brentford, United Kingdom, inhibits all three PHDs with a preference for PHD1 and PHD3 (Brigandi et al., 2016; Yeh et al., 2017). In June 2020, daprodustat received its first approval in Japan for the treatment of renal anemia (Dhillon, 2020). Multiple published studies have established the efficacy of daprodustat in managing anemia in CKD and improving iron metabolism (Johnson et al., 2014; Holdstock et al., 2016; Browe et al., 2019), which was noninferior to Erythropoiesis-stimulating agents (ESAs). The changes of iron parameters including hepcidin, ferritin and transferrin saturation were also observed as expected (Brigandi et al., 2016; Holdstock et al., 2016). Unfortunately, retinal hemorrhage and hypersensitivity (rash, dermatitis, urticaria), higher acceptance of antihypertensive medications with increasing systolic blood pressure, fatal and nonfatal myocardial infarction and heart failure exacerbations were reported in the daprodustat group (Meadowcroft et al., 2019; Akizawa et al., 2020b; Tsubakihara et al., 2020; Nangaku et al., 2021a). In two newest published studies (Singh et al., 2021a; Singh et al., 2021b), daprodustat showed a tumor effect in NDD patients while not in DD patients, which has not been reported before. Thus, more trials are awaited with interest and the safety and tolerability of daprodustat remain a problem for its clinical usage.

### 2.3 Vadadustat (AKB-6548)

Developed by Akebia, Cambridge, Boston, United States, vadadustat, an orally administered HIF-PHI based on a hydroxypyridine core and a carbonylglycine side chain, inhibits PHD3 more than the other two PHDs and stabilizes HIF-2α to a greater extent than HIF-1α (Maynard et al., 2003; Yu Y. et al., 2021). Studies published currently have proved an anemia-alleviating effect of vadadustat in CKD patients undergoing dialysis and non-dialysis with ESA-untreated or ESA-treated (Chertow et al., 2021; Eckardt et al., 2021; Nangaku et al., 2021c; Nangaku et al., 2021d). The drug has been approved in Japan for use in adult patients with anemia associated with CKD, and regulatory submissions are planned in the USA and the EU.

Several phase 3 multicenter trials showed that vadadustat was well tolerated and effective as darbepoetin alfa in maintaining Hb levels within the target range (Eckardt et al., 2021; Nangaku et al., 2021d). With regard to iron parameters, similar trends which were observed in roxadustat, such as decreases in serum ferritin, transferrin saturation, and hepcidin and an increase in total iron binding capacity, were shown in vadadustat treatment groups (Martin et al., 2017; Nangaku et al., 2020; Nangaku et al., 2021c). No evident changes have been observed in C-reactive protein (CRP) or total cholesterol (Martin et al., 2017; Haase et al., 2019). Gastrointestinal reactions, including nausea and diarrhea, were the most commonly reported drug-related adverse events in the vadadustat treatment arm (Pergola et al., 2016). Though the incidence of serious adverse events such as adverse cardiovascular events (either heart failure or thromboembolic events) in the vadadustat group lower than the darbepoetin alfa group (Eckardt et al., 2021), the underlying clinical implication of their occurrence was still unclear. Hypertension and hyperkalemia were also reported more frequently (risk ratio: 1.34 and 1.27, respectively) in the vadadustat group than in the placebo group. Because of the lack of great differences in blood pressure or electrocardiography in each group, further studied with more samples is in need to evaluate the safety of vadadustat (Pergola et al., 2016; Chen et al., 2021).

### 2.4 Molidustat (BAY 85-3934)

Molidustat, a once daily oral HIF-PHI evaluated by Bayer Health care, Leverkusen, Germany, inhibits all three PHDs, especially PHD3 (Bottcher et al., 2018; Akizawa et al., 2019a). In January 2021, molidustat was approved by the Pharmaceuticals and Medical Devices Agency for the treatment of renal anemia, and its molecular structure differs from other approved/late-stage PHD inhibitors in lacking a glycinamide side chain (Figg et al., 2021).

Several phase 3 studies reported a good response of molidustat in Japanese patients with renal anemia who were not treated with dialysis and who were undergoing hemodialysis or peritoneal dialysis (Macdougall et al., 2019; Yamamoto et al., 2019; Yamamoto et al., 2021). The endogenous EPO levels induced during treatment were close to the normal physiologic range of EPO, which proved its efficacy was non-inferior to ESAs (Akizawa et al., 2019c; Macdougall et al., 2019; Akizawa et al., 2021b). Molidustat has been shown to increase the availability of iron metabolism by the observation of changes in laboratory parameters (Macdougall et al., 2019), while the non-dropped levels of cholesterol and increasing levels of CRP may indicate a question of its safety (Akizawa et al., 2021b). In animal models, molidustat was shown to be effective in renal and inflammatory anemia, and unlike ESA therapy, it reduced blood pressure in an adenine-induced CKD model (Li L. et al., 2020). To date, though no adverse events of special interest have been reported, most of published trials have focused on its efficacy evaluation and no large-scale studies enrolled over 500 patients have been carried out. Further safety studies are warranted.

### 2.5 Enarodustat (JTZ-951)

The orally active HIF-PHI enarodustat developed by Japan Tobacco Inc., Tokyo, Japan was approved in September 2020 in Japan, and clinical development is ongoing in the United States and South Korea for the treatment of anemia associated with CKD. Enarodustat inhibits all three PHDs (Markham, 2021).

In preclinical studies, enarodustat has been found to increase HIF-α proteins, EPO production and erythropoiesis. Two phase 3 studies confirmed that its efficacy of correcting and maintaining hemoglobin levels was non-inferior to placebo and darbepoetin alfa in Japanese anemic patients with CKD not on dialysis or on maintenance hemodialysis (Akizawa et al., 2021a; Akizawa et al., 2019b). Enarodustat also shows no specific evidence on efficient iron utilization in iron-related parameters during erythropoiesis (Markham, 2021). The safety results in clinical trials demonstrate that enarodustat is generally well tolerated, with the most frequent adverse events being viral upper respiratory tract infection and gastrointestinal reactions (Akizawa et al., 2021a; Chen et al., 2021). Another phase III study estimating the efficacy and safety of this novel drug is underway in South Korea (NCT04027517), which helps to provide more reliable data for clinical use.

Taking the diversity of HIF target genes into consideration, HIF-PHIs have been evaluated for their potential in many non-anemic diseases, which would extend the clinical usage of HIF-PHIs to a much wider focus.

## 3 Potential Appliance of HIF-PHDs Inhibitors in Non-anemic Diseases

### 3.1 HIF-PHDs Inhibitors Protect Against Acute and Chronic Kidney Disease

**FIGURE 1:**
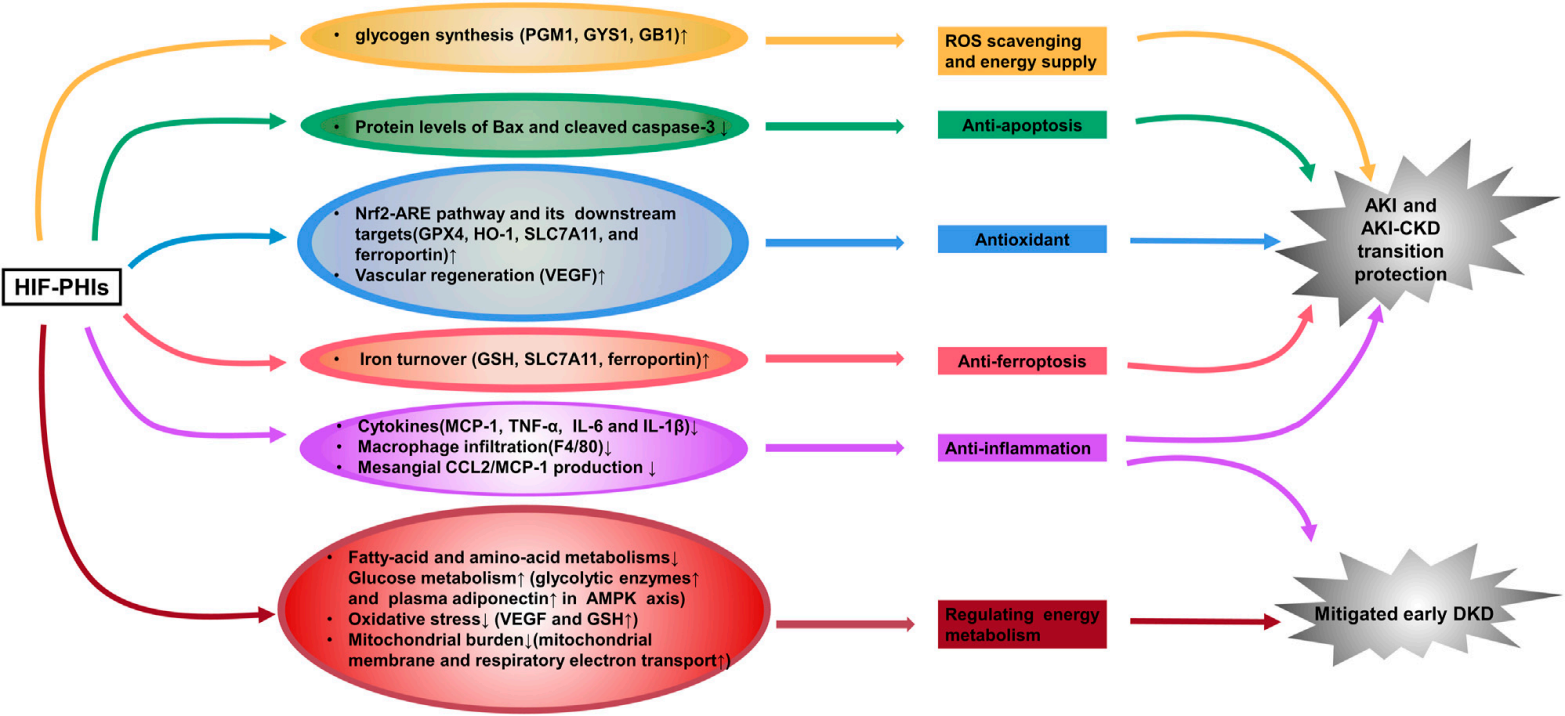
HIF-PHIs protect against acute kidney injury and incipient diabetic kidney disease. HIF-PHIs upregulate HIF expression in ischemia-, hypoxia- or toxication-induced AKI models and transactivate multiple target genes involved in anti-inflammation, anti-apoptosis, anti-oxidant, anti-ferroptosis, vascular regeneration and glycogen synthesis pathways to play a renal protective role. In diabetic kidneys, fatty acid and amino acid metabolism was upregulated, whereas HIF-PHIs downregulated these pathways and upregulated glucose metabolism. HIF-PHIs showed anti-inflammation effect, and counteract energy metabolism disorders, thus alleviating early diabetic kidney pathology. HIF, hypoxia inducible factor; HIF-PHIs, HIF prolyl hydroxylase domain enzyme inhibitors; AKI, acute kidney injury; CKD, chronic kidney disease; DKD, diabetic kidney disease; MCP-1, monocyte chemoattractant protein-1; TNF-α, tumor necrosis factor alpha; IL-6, interleukin-6; IL-1β, interleukin-1 beta; GPX4, glutathione peroxidase 4; HO-1, Heme oxygenase 1; SLC7A11, solute carrier family 7 (anionic amino acid transporter light chain, xc-system), member 11; VEGF, vascular endothelial growth factor; GSH, glutathione; PGM1, phosphoglucomutase-1; GYS1, glycogen synthase 1; GB1, 1,4-α glucan branching enzyme.

Ischemia and cellular toxicity are two major pathological factors consequently leading to acute kidney injury (AKI), which causes a rapid decline in oxygen tension and severe ischemic injury and thus deprives cellular energy and impairs physiological functions. To date, there are no effective therapies available clinically. Accumulating evidence demonstrates that roxadustat upregulates HIF expression in ischemia-, hypoxia- or toxication-induced AKI models and can activate multiple target genes to play a renal protective role. Our team first illustrated the blunt inflammatory and apoptotic response in roxadustat pre-treated AKI mice and cultured renal tubular epithelial cells induced by cisplatin, which was delineated as lower secretion of proinflammatory cytokines and decreased protein levels of Bax and cleaved caspase-3 (Yang et al., 2018). Coincidentally, Li X. et al. (2020) then reported that in addition to activating HIF-1α, roxadustat pretreatment enhanced nuclear factor erythroid 2-related Factor 2 (Nrf2) and decreased ferroptosis at the early stage of folic acid-induced acute kidney injury and retarded fibrosis progression afterward. More recently, two groups showed that roxadustat pre-treatment remarkably provided kidney injury relief in a mouse model of ischemia–reperfusion (I/R)-induced AKI through attenuation of inflammatory responses (decreased infiltration of macrophages and downregulated expression of inflammatory cytokines) and attenuated mitochondrial damage (increased ATPβ, PPARγ, mitochondrial DNA copy number, and decreased cytoplasmic cytochrome C) (Miao et al., 2021; Zhang et al., 2021). Besides Roxadustat, Ito et al. (2020) also found a kidney protection effect of enarodustat pre-treatment against ischemia injury and the mechanism involved an up-regulation of glycogen synthesis (Figure 1). However, currently no evidence has proven the therapeutic effect of HIF-PHIs against AKI when applying post injury. Nevertheless, since the onset time of potential AKI invoked by radiation, chemotherapeutic agents, and kidney donation and transplantation (when ischemia reperfusion injury occurs) is predictable, HIF-PHIs pre-treatment may be of help to avoid unnecessary damages under these conditions.

Besides AKI protection, our group proposed that 3 days post I/R injury administration of roxadustat showed great value in reversing the AKI-CKD transition (Wu et al., 2021). Similar attenuating effect of roxadustat was reported in an adenine-induced nephropathy model (Schley et al., 2019) (Figure 1). However, researchers see a dose- and time-dependent biphasic effect of HIF-PHIs on renal fibrosis (Yu et al., 2012; Li et al., 2019; Kabei et al., 2020). In a unilateral ureteral obstruction (UUO)-murine model, Kabei et al. (2020) found that a lower dose of roxadustat (12.5 mg/kg/day) had no effect on the mRNA expression of profibrogenic molecules regardless of the 3- or 7-day trial; while higher dose of roxadustat (50 mg/kg/day) remarkably increased such gene expression at 3 days but not 7 days. Similarly, in subtotal nephrectomy rat, early initiation (week 2–12) of a HIF-PHI L-mim treatment exacerbated renal dysfunction and fibrosis; L-mim treatment initiated in a more advanced stage of progression (week 4–12) predominantly preserved the peritubular capillary network and ameliorated the progression of CKD; and end-stage L-mim treatment (week 8–12) posed no effect (Yu et al., 2012). These finding was most consistent with the previous hypothesis that roxadustat may play a profibrotic role only at the early phase of renal fibrosis. But this influence faded away with the progression of tubulointerstitial fibrosis and even showed a protective reaction (Kabei et al., 2018). Current Phase 3 studies of CKD anemia for 3 of the leading HIF-PHIs have not been able to demonstrate a reduction in rate of progression of CKD as measured by eGFR, properly because the treatment was employed at advanced CKD stage (Chen et al., 2019b; Nangaku et al., 2021b; Singh et al., 2021a). Thus, to maximally and accurately utilize its ability to attenuate renal damage, timing of drugging is critical. More precise pre-clinical studies and clinical trials are needed to enlighten the underlying mechanism and take the initiative of using the HIF-PHIs to retard CKD progression.

Research on HIF-PHIs in diabetic kidney disease is now very limited. One recently published work demonstrated that enarodustat counteracts alterations in renal energy metabolism and mitigates urinary albumin excretion and renal pathological abnormalities (glomerulomegaly and glomerular basement membrane thickening) in incipient diabetic kidney disease, using streptozotocin-induced diabetic rats and alloxan-induced diabetic mice (Hasegawa et al., 2020) (Figure 1). The same group reported that enarodustat also exerted renoprotective effects against metabolic disorders and associated kidney disease in obese type 2 diabetic mice by improving glucose and lipid metabolism and suppression of CCL2/MCP-1 (Sugahara et al., 2020) (Figure 1). Consistently, an *in vitro* study carried out by Xie et al. (2019) showed less impairment brought by roxadustat treatment in high glucose-induced rat glomerular endothelial cells. However, the activity of HIF signaling in diabetic kidney is still controversy with one study reported tubule-specific knockout of HIF-1α aggravated kidney dysfunction and renal histopathological alterations in streptozotocin-induced diabetic mice (Jiang et al., 2020), and another elegant study, on the contrary, found that a specific inhibitor of HIF-1 attenuates the manifestations of diabetic nephropathy in OVE26 type 1 diabetic mice (Nayak et al., 2016). Moreover, the effect of HIFs on other renal resident cells under diabetic pathological conditions may be contradictory. Upregulation of HIFs activates VEGF signaling in renal glomerular endothelial cells and promotes angiogenesis. However, abnormal angiogenesis results in formation of immature new vessels, contributing to the development of capillary leakage and play a pathological role in diabetic kidney disease (Liu et al., 2018). Thus, there is a long way to go for the potential usage of HIF-PHIs in diabetic kidney disease.

### 3.2 HIF-PHDs Inhibitors in the Prevention of Retinopathy of Prematurity, Retinal Detachment and Meibomian Gland Dysfunction

In premature infants, oxygen supplementation not only acts as a life-sustaining measure to prevent mortality but also as a double-edged sword for its toxicity to retinal development. Retinovascular growth attenuation and vascular obliteration caused by hyperoxia-induced downregulation of HIF protein levels lead to retinopathy of prematurity (ROP), a disease accounting for over 100,000 new cases of infant blindness each year (Hartnett and Penn, 2012). Pharmacological activation of HIFs by roxadustat can prevent experimental oxygen-induced retinopathy (Sears and Hoppe, 2013) and thus has the potential to prevent blindness in children. Based on the mouse oxygen-induced retinopathy model conducted by Hoppe et al. (2016), two pathways have been convinced to be involved in the capillary bed protection effect of roxadustat against oxygen toxicity: one is stimulating the liver to secrete angiogenic hepatokines, such as EPO, PAI-1, orosomucosoid, adrenomedulin, apelin, VEGF, and angiopoietin-like protein 3. The other pathway is locally stimulating retinal protection. They demonstrated that roxadustat not only reduces capillary dropout in retinal flatmounts and induces normal and sequential retinovascular repair but also has the potential to reduce cell apoptosis and preserve retinal function, as evidenced by caspase 3 immunohistochemistry and electroretinography. Systematic biology further revealed the underlying metabolome mechanism of roxadustat’s retinovascular protection effect in hyperoxia (Singh et al., 2018). Performed by untargeted gas chromatography–mass spectrometry on retina samples collected in hyperoxia and on primary human retinal endothelial cells, researchers found that 3-hydroxypyruvate, an angiostasis metabolite, was over accumulated in response to hyperoxia and that roxadustat was able to stimulate the conversion of 3-hydroxypyruvate to serine, thus relieving the angiostasis effect. Then, they conducted an untargeted metabolite profiling using serum and retinal from newborn mouse pups exposed to phosphate-buffered saline or roxadustat, and pointed out that HIF stabilization by roxadustat acivates serine and 1-carbon metabolism (1CM). Inhibition of 1CM by methotrexate blocked roxadustat-mediated protection against ROP, demonstrating that increased serine/1CM participates in protection induced by roxadustat. Interestingly, isotopic tracing revealed that retinal serine is primarily derived from hepatic glycolytic carbon. This indicated that apart from the up-mentioned hepatic angiogenic hepatokines, serine act as an alternative signaling molecular to achieve the remote regulation of liver to retinol and mediated roxadustat’s retinovascular protection effect (Hoppe et al., 2016; Singh et al., 2019). Importantly, roxadustat demonstrated a weak induction effect on Müller cell HIF-2α, which is the main mediator of retina pathologic angiogenesis, thus guaranting the safety when using this drug in ROP (Hoppe et al., 2020).

**FIGURE 2:**
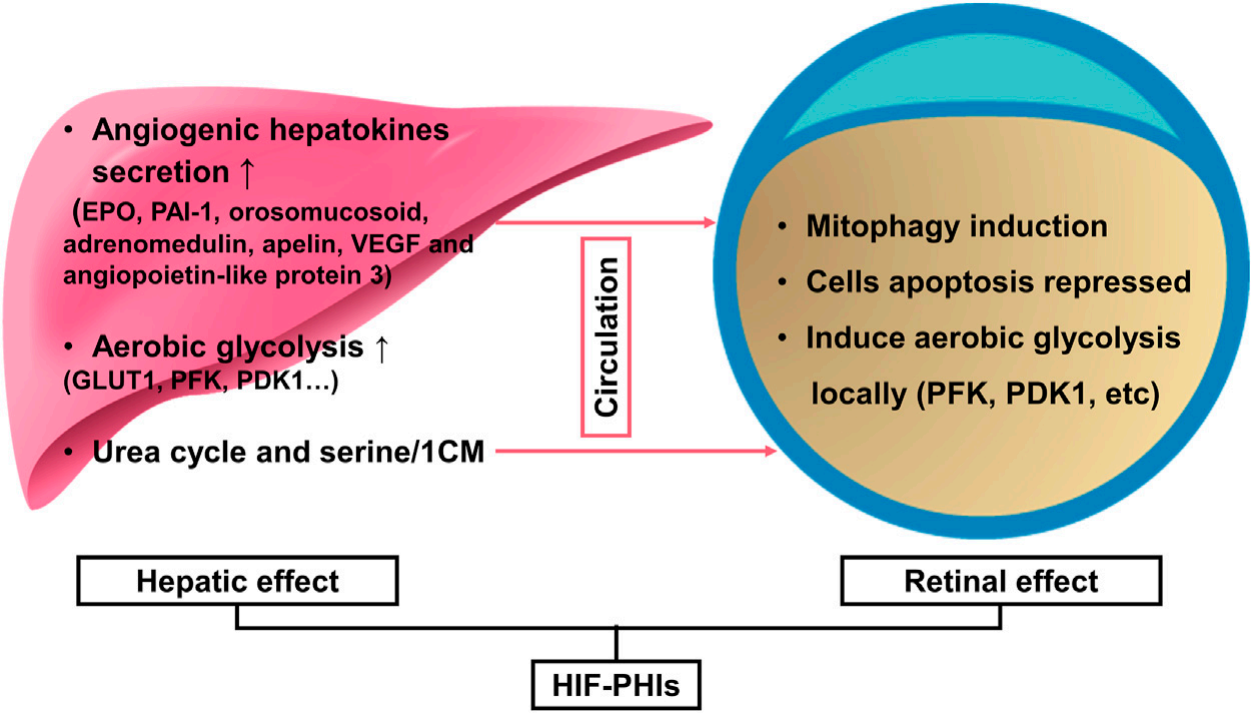
HIF-PHIs in the prevention of retinopathy of prematurity and retinal detachment. Together, liver remote protection and retinal local protection mediated the retinal protection effect of HIF-PHIs. On the one hand, systemic administration of HIF-PHIs invokes hepatic stabilization of HIF-1 and secretion of angiogenic hepatokines, such as EPO, PAI-1, orosomucosoid, adrenomedulin, apelin, VEGF and angiopoietin-like protein 3 thereafter. In addition, systemic HIF-PHI treatment dominantly upregulated the urea cycle and serine/1-carbon metabolism in the liver. The upregulated urea cycle and serine/1-carbon metabolism have proangiogenic effects and protect retinal blood vessels, thus emphasizing the importance of the liver in remote protection of the retina. On the other hand, HIF-PHIs enhance selective mitophagy against ROS, reduce cell apoptosis, and induce local aerobic glycolysis, which is reported to drive endothelial growth and repair. Thus, systemic HIF-PHI treatment targeting both the liver and the eye provides a rationale for protecting severe retinopathy of prematurity and retinal detachment. HIF-PHIs, HIF prolyl hydroxylase domain enzyme inhibitors; EPO, erythropoiesis; PAI-1, plasminogen activator inhibitor 1; VEGF, vascular endothelial growth factor; GLUT1, glucose transporter 1; PFK, phosphofructokinase; PDK1, pyruvate dehydrogenase kinase 1; 1CM, 1-carbon metabolism.

During hypoxia or ischemia, such as retinal detachment (RD), a specific target gene of the HIF-PHD pathway, BNIP3, was activated to protect the cell from cell death and induce mitochondrial autophagy (Zhang et al., 2008; Shelby et al., 2015). Such HIF-dependent expression is also known as selective mitophagy (Mazure and Pouyssegur, 2010). Liu et al. (2016) observed the phenomenon that retro-orbital injection of roxadustat could enhance such selective mitophagy against ROS injury to decrease photoreceptor cell death in experimental RD rats by reinforcing HIF-1 which displayed as the increasing pattern of the ratio LC3-II/LC3-I and the higher proportion of full-length autophagy-related protein 5 (Atg5) than cleaved Atg5. Additionally, in cultured comeal endothelial cells, mechanical stress during the perioperative period can be partly avoided by roxadustat preincubation (Bhadange et al., 2018), which indicates its potential in surgical trauma protection. In their *ex vivo* experimental study, damaging whole corneas with brief sonication resulted in an approximately 10% smaller injury area with 50 μM roxadustat and showed more vigorous protection than hypoxia. The effects of HIF-PHIs in ROP and RD are summarized in Figure 2.

In addition to retinopathy of prematurity and retinal detachment, Liu Y. et al. (2020) found the potential implication of roxadustat in meibomian gland dysfunction. The meibomian gland synthesizes and secretes a proteinaceous lipid mixture that enhances the stability of the tear film. Thus, meibomian gland dysfunction leads to a loss of meibum, destabilization and hyperevaporation of the tear film and results in dry eye disease. By stabilizing HIF-1α with roxadustat, the number of lipid-containing vesicles, the content of neutral lipids and the activity of DNase II were all increased in immortalized human meibomian gland epithelial cells. These findings suggest that local administration of HIF-PHIs may be beneficial for the treatment of meibomian gland dysfunction and relieve xerophthalmus. However, the protective effect of roxadustat on the meibomian gland needs to be verified *in vivo*, and the underlying mechanism also requires further investigation.

Thus, various mechanisms proved at different extent that HIF-PHIs showed retinal protection effect. Since ROP is predictable and the protection effect is mediated by liver and local retinal synchronously, it is suggested that HIF-PHIs administered systemically, starting at birth, continuing at 4–7 days intervals until 30 weeks, when the earliest stages of ROP can be seen (Hoppe et al., 2020). While in RD and meibomian gland dysfunction, local preparation might be more favorable, and it is important to institute the therapeutic dosage and duration for treating the disease without obvious systemic effects.

### 3.3 Potential of HIF-PHDs Inhibitors in Accelerating Bone and Tendon Regeneration

Nearly 10% of fracture cases face impaired healing (Holmes, 2017). A hallmark of impaired bone healing in humans and animals is a reduction in vascular supply and nutrient availability at the site of injury. Pharmacological activation of the HIF pathway by desferrioxamine (DFO) and L-mim was reported to upregulate VEGF expression and stimulate angiogenesis in primary mouse bone marrow mesenchymal stromal cells, human umbilical vein endothelial cells and explants of E17.5 mouse metatarsals, while HIF inactivation functions in the opposite way (Wan et al., 2008). Furthermore, by administering DFO into the distraction gap every 2 days from day 7 to 17 (period of active distraction), significantly increased vessel number and vessel connectivity in the mouse distraction osteogenesis model were noticed after the final injection. At day 31, increased bone regeneration was also observed in DFO treatment group (Wan et al., 2008). These results suggest the feasibility of HIF-PHIs in enhancing bone regeneration.

In addition, benefiting from its easy accessibility and highly expandable potential, mesenchymal stem cells (MSCs) are an ideal cell source to regenerate damaged cartilage and tenocytes; however, MSCs have the proclivity to undergo hypertrophic or non-specific differentiation. Roxadustat was reported to enhance chondrogenesis and attenuate hypertrophy dose-dependently *via* the PTHrP-PTHR1-MEF2C axis in cultured MSCs (Browe et al., 2019). Likewise, roxaduatat also enhanced the differentiation of adipose-derived MSCs (ADMSCs) to tenocytes in a coculture system consisting of human ADMSCs and tenocytes derived from adult female Sprague–Dawley rats, and this directional differentiation covered the shortage of ADMSCs (Yu et al., 2016). However, further work is required to test the potential *in vivo*. Compared to the long-term administration in renal diseases with confusing time and duration of interventions, the use of HIF-PHIs seems to be potentially more tangible in promoting bone and tendon regeneration.

### 3.4 Protective Role of HIF-PHDs Inhibitors in Cardiovascular Diseases

Ischemic injury heart disease (IHD) is a leading cause of heart failure. Pharmacological preconditioning with roxadustat could switch metabolism from aerobic to anaerobic respiration and produce ischemic tolerance, as shown by the reduction of infarct area (IFA), IFA per area at risk (IFA/AAR), plasma creatinine kinase activity and deceased percentage of cardiac TUNEL-positive cells in a murine cardiac I/R model (Deguchi et al., 2020). However, the nonparallel enhancement of aerobic respiration and the reduction of anaerobic respiration when HIF-1α was silenced reflected that other mechanisms may also account for roxadustat’s protective effect in IHD. In addition to roxadustat, Coyle et al. (2021) found that molidustat treatment increased the survival rate of cardiac organoids by improving endothelial expression (CD31) and lumen formation when exposed to both hypoxic and ischemic conditions.

Atherosclerosis is one of the major contributors to chronic heart diseases. Excitingly, evidence indicated that roxadustat might function to protect against obesity-induced atherosclerosis. In western diet-fed apolipoprotein E knockout (Apoe^−/−^) mice, roxadustat was able to stabilize adipose HIF-2α and intensify downstream alkaline ceramidase 2 (ACER2)-triggered ceramide catabolism. As ceramide is one of the atherogenic mediators, decreased ceramide resulted in decreased total cholesterol levels in plasma and decreased very low density lipoprotein (VLDL) cholesterol and LDL cholesterol levels in adipocytes (Zhang X. et al., 2019). Moreover, FG4497, an analog of roxadustat, showed a similar atherosclerosis protection effect (Rahtu-Korpela et al., 2014; Rahtu-Korpela et al., 2016). These results indicate the potential usage of HIF-PHIs as an effective therapeutic strategy for treating atherosclerosis. However, the clinical data so far has not shown a beneficial effect and perhaps much longer studies are needed (Martin et al., 2017; Haase et al., 2019; Akizawa et al., 2021b).

**FIGURE 3:**
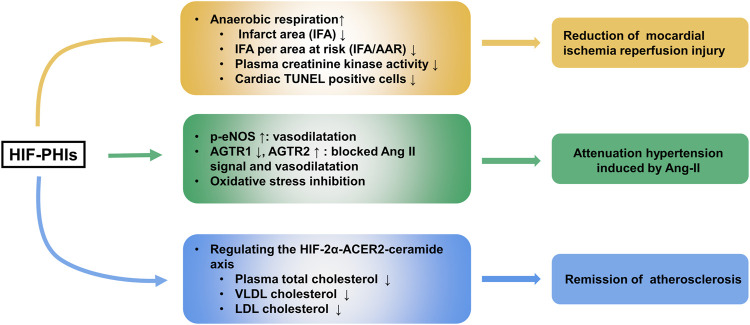
Protective role of HIF-PHIs in cardiovascular diseases. HIF-PHIs can switch metabolism from aerobic to anaerobic respiration and produce ischemic tolerance, thus protecting against ischemic injury in heart disease. Additionally, HIF-PHIs could remarkably ameliorate Ang II induced hypertension, possibly by stabilizing HIF-1α and subsequently targeting eNOS, AGTR1, AGTR2, and oxidative stress, indicating that HIF-PHIs could be explored as a treatment for hypertension associated with high RAS activity or eNOS defects. Moreover, roxadustat was able to stabilize adipose HIF-2α and intensify downstream ACER2-triggered ceramide catabolism. As ceramide is one of the atherogenic mediators, decreased ceramide resulted in decreased total cholesterol levels in plasma and decreased VLDL cholesterol and LDL cholesterol levels in adipocytes, indicating the potential usage of HIF-PHIs as an effective therapeutic strategy for treating atherosclerosis. HIF-2α, hypoxia inducible factor 2 alpha; HIF-PHIs, HIF prolyl hydroxylase domain enzyme inhibitors; IFA, infarct area; IFA/AAR, IFA per area at risk; eNOS, endothelial nitric oxide; Ang II, angiotensin II; AGTR1, Ang II Receptor Type 1; AGTR2, Ang II Receptor Type 2; ACER2, alkaline ceramidase 2; VLDL, Very-low density lipoproteins; LDL, low density lipoproteins.

The prevalence of hypertension is increasingly high worldwide, and it brings about a series of clinical complications, including cardiovascular diseases and systematic organ injuries, contributing to increases in morbidity and mortality (Lim et al., 2012). Several scientists are worried about the negative effects of HIF-PHIs on blood pressure due to erythrocytopoiesis and vascular regeneration (Wu et al., 2017). Our laboratory first reported that roxadustat alleviated Angiotensin II and L-NAME (an inhibitor of NO production) induced hypertension and associated organ injury (Yu J. et al., 2021). Our data showed that under roxadustat administration in murine Ang II-induced hypertension models, the expression of Ang II Receptor Type 1 (AGTR1), which impacts constricting vessels and reabsorbing salt and water, was decreased, while the expression of both AGTR2, a receptor that shows the opposite function to AGTR1, and endothelial nitric oxide (eNOS), which regulates the vascular tone of the small artery, was upregulated. In animal models, molidustat also showed blood pressure lowering effect in an adenine-induced CKD model (Li L. et al., 2020). However, clinical trials did not show an advantage of HIF-PHIs over regulating blood pressure in CKD patients either with or without dialysis (Barratt et al., 2021; Chen et al., 2021). Daprodustat even showed a hypertensive effect at high doses (attributed to a rapid rate of rise of Hb) (Meadowcroft et al., 2019). Thus, the effect of HIF-PHIs on blood pressure appears to be controversial and more investigations are expected to better clarify the role of HIF-PHIs in different types of hypertension. The effects of HIF-PHIs in ischemic heart disease, atherosclerosis and Ang II induced hypertension are summarized in Figure 3.

However, several academics have suggested that on account of the shift to inefficient glycolytic metabolism and mitochondrial biogenesis, the upregulation of HIF-1a leads to lipid accumulation and heart contractile dysfunction and thus may predict a poor prognosis in patients with chronic heart failure (Krishnan et al., 2009; Wei et al., 2016; Packer, 2020). Instead, HIF-2α accumulation is accompanied by ventricular remodeling, which has a protective effect (Jurgensen et al., 2004). Therefore, selective HIF-2α stabilization may account for a better strategy. Since selective inhibitors are still lacking, combining HIF-PHIs and HIF-1a inhibitors may selectively stimulate the expression of HIF-2α and eliminate the untoward effect of HIF-1a.

### 3.5 Neuroprotection Effect of HIF-PHDs Inhibitors

Previously, preischemic treatment with GSK360A and FG2216 was shown to decrease the infarct volume and improve behavior in rodent models of focal cerebral ischemia (Chen et al., 2014; Zhou et al., 2017). Elevated expression of EPO and a protective effect on blood brain barrier integrity may account for the observed protective effect. By measuring LDH release and the rising trend of cell viability in both oxygen- and glucose-deprived PC-12 cells and primary rat neurons, Singh et al. (2020) illustrated that other HIF-PHIs (roxadustat, daprodustat and molidustat) also exhibited neuroprotective effects and that the protective role may be autophagy-mediated.

Later research characterized the antiapoptotic effect of roxadustat in protecting against spinal cord injury (Wu et al., 2016). They found that roxadustat treatment significantly inhibited tert-butyl hydroperoxide-induced apoptosis and increased the survival of neuronal PC-12 cells with the downregulation of Bax and cleaved caspase-3 and upregulation of B-cell lymphoma-2 (Bcl-2). Additionally, roxadustat administration post spinal cord injury improved recovery and increased the survival of neurons in their mouse model, with higher motor rating scores and more motor neurons observed in the roxadustat-treated group than in the untreated group. This *in vivo* and *in vitro* effect was dependent on HIF-1 activation, since the protective effect was offset by the synergistic use of YC-1, a selective inhibitor of HIF-1.

**FIGURE 4:**
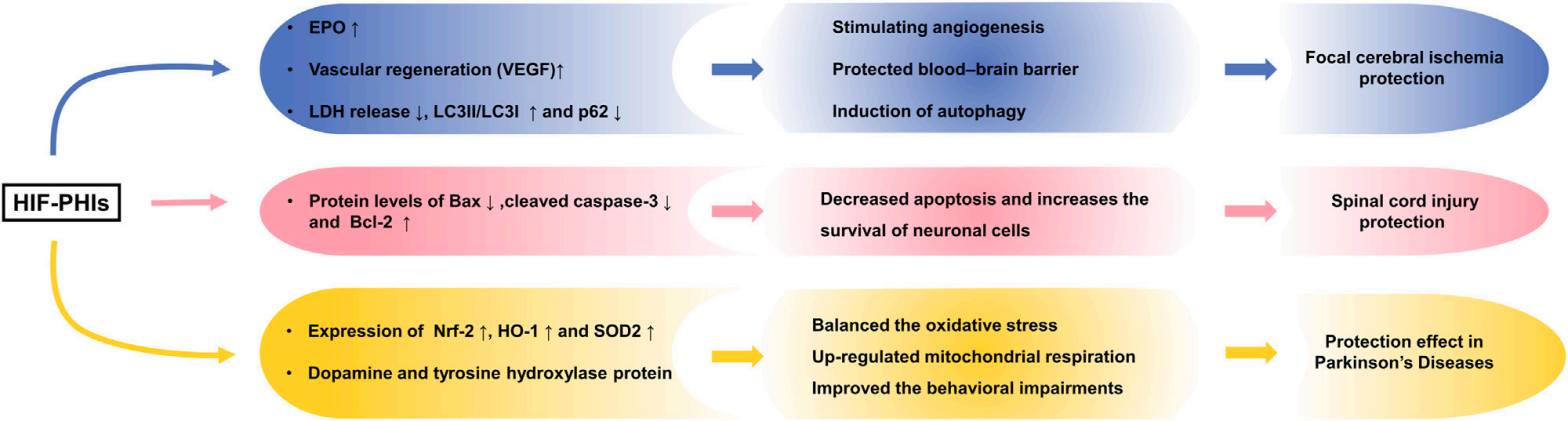
Neuroprotection effect of HIF-PHIs. Preischemic treatment with HIF-PHIs has been shown to protect against cerebral ischemia *in vivo* and *in vitro via* elevation of EPO, protection of the blood brain barrier, and autophagy activation of neurons. HIF-PHI treatment significantly inhibited apoptosis and increased the survival of neuronal cells with the downregulation of Bax and cleaved caspase-3 and upregulation of Bcl-2 in spinal cord injury. Additionally, HIF-PHI treatment upregulated mitochondrial respiration and counterbalanced oxidative stress by upregulating Nrf-2, HO-1 and SOD2 and rescuing the loss of dopamine and TH protein, showing improved behavioral impairments in Parkinson’s disease. HIF-PHIs, HIF prolyl hydroxylase domain enzyme inhibitors; EPO, erythropoiesis; LDH, lactate dehydrogenase; Bcl-2, B-cell lymphoma-2; LC3, light chain 3; Bax, Bcl-2 associated X protein; Nrf2, nuclear factor erythroid-2-related factor 2; HO-1, Heme oxygenase-1; SOD2, Superoxide dismutase 2.

This finding of neuroprotection has been applied to the study of Parkinson’s disease (PD). In one original study, Li et al. (2018) reported the neuroprotective effect of roxadustat in PD. PD is a neurodegenerative disorder mainly characterized by deficiency of the neurotransmitter dopamine and abundant tyrosine hydroxylase (TH) in the striatum of the brain, and 1-methyl-4-phenyl-1,2,3,6-tetrahydropyridine (MPTP) is known as a neurotoxin that impairs dopaminergic neurons. Specifically, roxadustat reversed MPTP-induced SH-SY5Y neuroblastoma cell apoptosis, upregulated mitochondrial respiration and counterbalanced oxidative stress by upregulating Nrf-2, heme oxygenase-1 (HO-1) and superoxide dismutase 2 (SOD2). Also, preconditioning with roxadustat in MPTP-treated mice rescued the loss of dopamine and TH protein in the striatum and partially improved behavioral impairments. These results demonstrated that roxadustat is a promising therapeutic strategy for PD and that the protective effect may rely on mitochondrial function improvement (Iyalomhe et al., 2017). The neuroprotective effects of HIF-PHIs are summarized in Figure 4.

It is worth noting that existing reported neuroprotection effect of HIF-PHIs is mostly been set as pre-emptive treatment. Since cerebral ischemia injury and spinal cord trauma often happens suddenly, pre-treatment is obviously impractical in clinic. Thus, whether HIF-PHIs can still play its protective role when applying post injury remains to be proven. What’s more, whether HIF-PHIs can still play its protective role in other pathological settings also required more investigation, since in several models of traumatic brain injury *in vivo* and vitro (Bae et al., 2018), highly expressed HIF-1α transactivated the expression of leucine-rich repeat kinase 2, and exacerbated neuronal cell death following injury.

### 3.6 HIF-PHDs Inhibitors in Wound Healing and Tissue Transplantation

Stabilization of HIF-1α has been widely reported to be a critical factor in the improvement of wound healing (Zhu et al., 2020). Olson et al. (2019) reported improvements in wound healing by evaluating the wound area, volume and depth in daprodustat topical formulation-treated healthy volunteers with intact skin and diabetic foot ulcer patients and the mechanism was illustrated by activation of HIF-1 signaling and promoted vascularization. Zhu et al. (2019) reported a similar effect of roxadustat on wound healing in diabetic rats. In a more recently published study, the authors report the design and synthesis of cyclometalated iridium (III) metal complex 1a as a stabilizer of HIF-1α and its promotion effect in accelerating wound healing. According to their findings, in addition to HIF-1α/VEGF, wound healing-related genes such as heat shock protein-90, VEGFR-1, stromal cell-derived factor-1, stem cell factor, and Tie-2 are also increased in the wound tissue by local administration of complex 1a in diabetic mouse models (Li G. et al., 2021). In addition to local treatment with HIF-PHIs, HIF-1-activated bone marrow-derived angiogenic cell infusion was also reported as an effective combination strategy in improving burn wound healing in aged mice (Du et al., 2013).

Angiogenesis improvement was also conceived as a possible solution to improve the survival rate of tissue transplants. In the Fisher–Lewis rat model of allogenic kidney transplantation, Bernhardt et al. found that donor pretreatment using a single dose of a small molecule inhibitor of FG-4497, an analog of roxadustat, significantly reduced the frequency of delayed graft function and markedly improved long-term outcome (Bernhardt et al., 2009). Similarly, pharmaceutical stabilization of HIF-1 with roxadustat in cardiac death donors significantly improved graft liver function with increased bile production and synthesis of ATP and decreased liver enzyme release, histology injury scores and oxidative stress-induced cell injury and apoptosis after reperfusion in a rat model (Zhang et al., 2018). By using the rat subcutaneous chamber model, Zhou et al. found that loading roxadustat exhibits an increasing thickness of fibrovascular tissue and a larger average diameter of vessel tubules, which indicated that the beneficial effect of HIF-PHIs in maintaining tissue transplants is thought to promote the maturation of neovascularization (Zhou et al., 2019). Another strong piece of evidence presented by Luo et al. (2019) showed that roxadustat obviously relieved H_2_O_2_-induced apoptosis and promoted the survival of PC-12 cells and bone marrow-derived stem cells, which provides a promising candidate for improving the success rate of bone marrow transplantation in spinal cord injury. Since it is feasible to take pre-treatment of donors before organ harvesting for transplantation, HIF-PHIs can be employed to optimize preservative and increase survival rate of organs.

### 3.7 Antineoplastic Activity of HIF-PHDs Inhibitors and Their Organ Protection Effect Under Radiation Treatment and Chemotherapy

Adaptive responses to hypoxia are involved in the progression of cancer. As tumors expand, lack of oxygen results in the activation of the hypoxia response. Thus, for most tumor microenvironments, HIF stabilization and the VEGF stimulation effect of HIF-PHIs is a contradictory therapeutic plan in tumors. However, Nishide and his coworkers found that in Lewis lung carcinoma and B16F10 melanoma tumor models, roxadustat exhibited a significant dose-dependent inhibition of tumor growth, and this amazing impact relied on neither apoptosis activation nor inhibited proliferation but on phagocytosis activation in macrophages and normalization of tumor vessels and the tumor microenvironment (Nishide et al., 2019). In addition to roxadustat, Nishide et al. (2020) proved that 3 more PHD inhibitors, daprodustat, molidustat, and vadadustat, also function similarly, although each of them displayed their own different priority of gene expression profiles. Kachamakova-Trojanowska et al. (2020) confirmed the effectiveness of molidustat in tumor supression in cultured MDA-MB-231 breast cancer cells and MDA-MB-231 xenograft *in vivo*. Consistently, in another elegant study, Price et al. (2019) found that genetic ablation of PHD2 or roxadustat administration can both inhibit the proliferation of a subset of clear cell ovarian cancer and melanomas. To note, the antineoplastic activity of HIF-PHIs may be limited to only a small subset of tumors. However, given the function of PHDs in cancer models is incompletely understood, the involvement of diverse HIF-PHIs will further complicate the picture. More in-depth studies are awaited to test the responsiveness to therapy of different HIF-PHIs on various types of tumor.

In addition to antineoplastic activity in a certain analog of tumors, HIF-PHIs showed an organ protective role against non-targeted radiation and chemotherapy. Doxorubicin, an effective chemotherapeutic agent applicable to diverse cancers, is limited clinically due to its cardiotoxicity. Our team previously found that roxadustat counterbalanced doxorubicin-induced mitochondrial oxidative stress in cardiomyocytes by increasing the expression of HIF-1a and its target genes SOD2 and Bcl-2, which finally blocked the apoptosis of cardiomyocytes and protected heart function (Long et al., 2020). Radiation therapy is the only accessible method for locally advanced or metastatic pancreatic carcinoma, but its lethal gastrointestinal toxicities are quite worrying. Fujimoto and his colleagues (Fujimoto et al., 2019) measured increasing microvessel density and quantified less epithelial apoptosis in roxadustat-treated K-ras; Trp53; Pdx-1-Cre(KPC) mice, a widely used transgenic pancreatic ductal adenocarcinoma model, which suggested that roxadustat is an effective remedy in improving the survival rate and maintaining intestinal function during radiation therapy. In addition, FG-4497 also functions to increase hematopoietic stem cell (HSC) quiescence and enhance HSC survival in the bone marrow of irradiated mice, as measured in long-term competitive repopulation assays, which efficiently alleviates marrow suppression (Forristal et al., 2013).

### 3.8 HIF-PHDs Inhibitors in Improving Obesity and Other Metabolic Disorders

Adipose tissue functions not only to reserve fat but also as an endocrine organ and functions widely in the whole body. Adipose tissue macrophages play a key role in mediating proinflammatory responses in the adipose tissue, which is associated with insulin resistance. Latest report found that FG-4592 perhaps ameliorated macrophage inflammasome activation in mice fed with high-fat diet (HFD), and protected against insulin resistance and obesity symptoms (Li X. et al., 2021). Moreover, Saito et al. (2019) showed that in mice fed with HFD, JTZ-951 effectively decreased macrophage infiltration into white adipose tissue and protected against obesity-related liver steatosis, and kidney injury as evidenced by decreased macrophages infiltration, mesangial expansion and reduced albuminuria. Thus, HIF-PHIs were shown to lower the risks of obesity-related diseases by protection against insulin resistance via remission of inflammation in adipose tissue and prevention from organ impairment related to obesity.

In roxadustat-treated high-fat diet murine models (Zhang X. et al., 2019) and in various clinical trials (Chen et al., 2019b; Anker et al., 2020), decreased cholesterol levels were also observed. Furthermore, roxadustat also ameliorated liver steatosis, improved liver histology and even reduced copper accumulation in models with the ATP7b mutant (Mi et al., 2020), a key reason leading to Wilson’s disease, while the underlying mechanism needs to be delineated in the future.

In addition to lipid metabolism, various published clinical trials observed a reduction in hepcidin, a hormone responsible for iron homeostasis, and mobilization of internal iron stores in response to roxadustat. Del Balzo et al. (2020) quantified lower levels of hepcodon mRNA expression in the liver and higher levels of the iron transporter Slc11a2 and duodenal cytochrome b in the duodenum of a roxadustat-treated rat model of anemia induced by peptidoglycan-polysaccharide. These results suggested that the novel drug is superior to traditional ESA in easing functional iron deficiency in anemia models. Noonan et al. (2020) proposed the hypothesis of the relationship between iron utilization and fibroblast growth factor-23 (FGF23) production, a vital regulator of phosphate homeostasis. In their CKD mouse model fed with adenine-containing diet, the roxadustat cohort showed more marked lowering of FGF23 production and increasing vitamin D 1α-hydroxylase (Cyp27b1) expression compared to the EPO cohort, greatly rescuing the imbalanced mineral metabolism and opening up a new avenue for therapy of hyperparathyroidism and metabolic bone diseases.

### 3.9 HIF-PHDs Inhibitors in Protection of Respiratory Disease

Sepsis is one of the life-threatening causes of sudden deaths of patients in the intensive care unit and usually starts from lung collapse first. Han et al. (2020) found alleviated inflammatory cell infiltration and relieved lung necrosis by roxadustat in LPS-induced septic mice (roxadustat injected right after LPS treatment).

Idiopathic pulmonary fibrosis is the most common idiopathic interstitial pneumonia with poor prognosis and limited treatment strategies. Huang et al. (2020) first proposed the attenuation effect of roxadustat on pulmonary fibrosis in CoCl_2_-stimulated mouse lung fibroblast (L929) cells and a bleomycin-induced pulmonary fibrosis mouse model. They demonstrated that roxadustat suppressed cell proliferation and the expression of collagen I, collagen III, α-SMA, TGF-β1, CTGF and p-Smad3 both *in vitro* and *in vivo*. In addition, when SB525334 (an inhibitor of TGF-β1 activation) or SIS3 (an inhibitor of Smad3) was added, the cell proliferation rate and protein expression levels were no further lower than before. All these results suggest that roxadustat functions to reduce collagen fiber formation and deposition *via* the TGF-β1/Smad3 pathway, thus improving lung coefficients and histopathological lesions in lung tissues. In a mouse model of bronchopulmonary dysplasia (Hoppe et al., 2016), instead of destructive or enlarged alveoli, roxadustat normalized alveolar size, showing great alveolar protection and repair ability.

However, Cygulska et al. (2019) documented a case in which a 74-year-old patient developed severe pulmonary arterial hypertension when taking part in a phase 3 clinical trial of roxadustat therapy for anemia, and later conditions improved after this drug was discontinued. The authors infer that by stabilizing HIF-2α, roxadustat upregulates not only Notch3 and TGF-β (Wang et al., 2016) but also EPO (Karamanian et al., 2014), which stimulates the proliferation of pulmonary endothelial and smooth muscle cells and probably further leads to the remodeling of pulmonary vasculature during pulmonary arterial hypertension development. For clinicians, such severe adverse reaction should be taken into consideration when applying HIF-PHIs clinically.

### 3.10 Potential Usage of HIF-PHDs Inhibitors in the Prevention and Treatment of COVID-19

HIF is reported to be induced in host cells confronted with bacterial, viral, protozoal, and fungal infections. In turn, after being stabilized or activated, HIF participates in innate and adaptive immune responses and influences the outcome of the infection. The innate immune response starts with the epithelial barrier and is reinforced by phagocytic cells and T cells. Stabilized HIF in epithelial cells and dendritic cells can upregulate the expression and release of chemokines, which could recruit neutrophils to the inflammation site. Meanwhile, HIF enhances the migration of neutrophils and macrophages to the site of infection, reduces apoptosis and increases the retention of both types of cells at the site of tissue injury (Kong et al., 2004; Elks et al., 2011). However, HIF-1a negatively regulates the activity of T cells by reducing T cell survival and proliferation, which play an important role in adaptive immune responses (Larbi et al., 2010). Thus, the complex and cell-specific roles of HIF render it difficult to predict the net effect of HIF-PHIs *in vivo*, and detailed research should be carried out under each condition.

Excitingly, roxadustat showed a potential retarding effect on the ongoing coronavirus SARS-CoV-2. SARS-CoV-2 primarily infects lung epithelial cells by binding to angiotensin-converting enzyme 2 (ACE2). Amazingly, roxadustat was reported to reduce the mRNA and protein levels of ACE2 expression across a range of cell lines and in mouse lung tissue, thus inhibiting the entry of SARS-CoV-2. Consistently, in a recent published paper, the researchers reported an HIF-1a-dependent induction of the microRNA LET7b, which then directly suppresses the expression of ACE2 in hypoxic induced pulmonary artery smooth muscle cells (Zhang R. et al., 2019). Thereafter, in addition to its effects on ACE2-mediated viral entry, HIF-PHIs mediated the suppression of SARS-CoV-2 RNA replication and secretion of infectious particles post entry. This is not surprising since HIF has been shown to suppress the replication of other RNA viruses through modulating host cell metabolism (Farquhar et al., 2017; Zhao et al., 2020). This raises the potential use of HIF-PHIs in the prevention and treatment of COVID-19 (Wing et al., 2021). Since substantial numbers of CKD anemia patients currently using roxadustat or other HIF-PHIs are at risk of infecting SARS-CoV-2, monitor these patients for any evidence of prophylactic or therapeutic activity against COVID-19 will be helpful for the clinical translation of these drugs.

## 4 Discussion

Hypoxia have been associated with a number of pathological conditions, while HIFs are the main transcriptional factors tuning gene expression and orchestrating cellular function. The critical role of HIFs makes it an ideal target for small molecule intervention. Thus, the chemical scientist has been working on inhibiting of PHDs for decades aiming to boost the expression of HIF with no obvious toxicity. Taken the reliable efficacy and safety, roxadustat has finally taken lead in receiving formal market authorization. Daprodustat, vadadustat, moliduustat and other novel inhibitors are in clinical trials as well.

CKD is an increasingly public health challenge with a prevalence of 11%–13% worldwide (Hill et al., 2016). In chronic kidney disease, EPO production is usually in a disruption state, leading to a decrease in Hb (Hb < 13 g/dl for men and <12 g/dl for women) (Coresh et al., 2007), labeled as renal anemia. The clinical availability of HIF-PHIs is like opening a new era for the management of renal anemia. By stabilizing HIFs, HIF-PHIs ameliorate anemia via increasing the expression level of HIF target gene EPO and improving iron utilization efficiency. There are several advantages of PHD inhibitors over conventional ESAs: lower cost, more convenient administration (oral administration) and better compliance, physiological levels of endogenous EPO production and less cardiovascular and cerebrovascular events, accompanied by improved iron utilization efficiency.

As HIF is involved in many physiology and pathology conditions, efforts have been made to extend the potential usage of HIF-PHIs beyond CKD anemia. This paper has reviewed the progress of pre-clinical and clinical research regarding to HIF-PHIs in different organs and systems. However, it can also be classified according to acute tissue injury caused by hypoxia, toxins or irradiation, wound healing and tissue transplantation, chronic tissue injury and fibrosis, metabolism disorder, inflammation disease and pathogen infection, etc. Pretreatment of HIF-PHIs showed a protective role in acute tissue injury, and the mechanism is mainly dependent on their apoptosis inhibition, and anti-oxidative and energy metabolism resetting effect. In the process of wound healing, bone regeneration and tissue transplantation, HIF-PHIs functions to accelerate the process by promoting HIF/VEGF induced angiogenesis. The increased vascular density thus guarantees the supply of oxygen and nutrient at the site of injury. Besides, HIF-PHIs also exert retinopathy protection effect via hepatic and local loops.

However, the effect of HIF-PHIs is much more controversy in the condition of chronic tissue injury and fibrosis, as well as in infectious diseases. It seems that the effect of HIF-PHIs on renal fibrosis is dose and time dependent, similar phenomenon might also exist in IPF. HIF-PHIs enhances the anti-pathogen ability of epithelial cells, dendritic cells and neutrophils while negatively regulates the activity of T cells. Thus, the complex and cell-specific roles of HIF also render it difficult to predict the net effect of HIF-PHIs on pathogen *in vivo*. So is the effect of HIF-PHIs on sterile inflammation owing to physical, chemical and metabolic stressors. Miao et al. (2021) found that roxadustat could suppress the release of inflammatory factors in the model of renal I/R-induced injury mice whose observation period is 2 days. While our group found roxadustat posed no effect on the level of TNF-α and IL-6 in serum and heart of doxorubicin-induced mice (Long et al., 2020; Wu et al., 2021). In experimental colitis, Higashiyama et al. (2012) reported that stimulation of HIF-1 exacerbates inflammatory cell infiltration in experimental colitis. The effect of HIF-PHIs on sterile inflammation seems to be tissue or context dependent.

Besides the pleiotropic role of HIF, another important question is that HIF is not the only substrate of PHD enzymes. Multiple potential non-HIF substrates of the PHD enzymes have been reported. PHDs altered the expression of Akt (Guo et al., 2016), centrosomal protein Cep192 (Moser et al., 2013) and FOXO3a (Zheng et al., 2014) to regulate cell proliferation and viability, and showed their potential contribution to tumor development and metastasis via the changes of F-actin (Luo et al., 2014), NFκB (Cummins et al., 2006) or Sprouty2 (Anderson et al., 2011). PHD was reported to respond to nutrient deprivation through hydroxylation of B55α (Di Conza et al., 2017) or ACC2 (German et al., 2016). What’more, organ function maintainence in liver, lung, cardiac and even neurons via up-/down-regulating the levels of actin cross-linker filamin A (Segura et al., 2016), β(2)-adrenergic receptor (Xie et al., 2009), phosphodiesterase 4D (Huo et al., 2012) and thyroid hormone receptor-α (Xie et al., 2015) was also reported to be controlled by PHDs. These potential PHD substrates might transduce cellular responses other than hypoxia sensing and adapting and generate off-target effects of PHD inhibitors. We also raised the possibility if PHDs participate physiological effects other than acting as hydroxylase enzymes. More work should be carried out to clarify the multiformity of PHDs and HIF-PHIs.

## 5 Conclusion and Future Perspective

In conclusion, HIF-PHIs roxadustat, daprodustat, vadadustat, molidustat, and enarodustat have been approved for clinical usage or progressed into clinical trials for anemia treatment currently. As HIF is involved in many physiology and pathology conditions, efforts have been made to extend the potential usage of HIF-PHIs beyond CKD anemia. This paper has reviewed the progress of pre-clinical and clinical research regarding to HIF-PHIs in different organs and systems. HIF-PHIs pre-treatment may be of great help in practical to avoid unnecessary damages in chemotherapeutics or irradiation induced organ damage and organ donors and recipients for optimizing preservative and increasing survival rate and long-term function of organs. Local HIF-PHIs administration will be beneficial in retinal detachment, meibomian gland disfunction, wound healing, and bone and tendon regeneration. In ischemic heart disease, focal cerebral ischemia, spinal cord injury where trauma often happens suddenly, although pre-emptive treatment with HIF-PHIs showed protective effect, whether HIF-PHIs can still play the protective roles when applying post injury remains to be proven. Moreover, HIF-PHIs showed potential in attenuating atherosclerosis, hypertension, Parkinson’s disease, obesity and other metabolic disorders. However, due to the potential invoke of polycythemia, pulmonary artery hypertension and possibly occurrence of cancer, pre-clinical and clinical research aiming to guide the proper dosage and frequency of the pharmacological administration under these pathological conditions that need long term treatment are urgently awaited. The effects of HIF-PHIs are much more controversy in the condition of chronic tissue injury and fibrosis, cancer and infectious diseases, warranting in-depth research.

## Glossary

1CM: 1-carbon metabolism
ACER2: Alkaline ceramidase 2
ADMSCs: Adipose-derived MSCs
AGTR1: Ang II Receptor Type 1
AKI: acute kidney injury
Atg5: Autophagy-related protein 5
Bcl-2: B-cell lymphoma-2
BNIP3: BCL2/adenovirus E1B 19 kDa protein-interacting protein 3
CKD: chronic kidney disease
CRP: C-reactive protein
DD-CKD: Dialysis-dependent (DD)-CKD
DFO: Desferrioxamine
eNOS: endothelial nitric oxide
EPO: erythropoiesis
ESAs: erythropoiesis stimulating agents
FGF23: Fibroblast growth factor-23
FIH: factor-inhibiting HIF
Hb: hemoglobin
HFD: high-fat diet
HIF-PHIs: HIF-PHDs inhibitors
HIFs: hypoxia inducible factors
HO-1: heme oxygenase-1
HSC: hematopoietic stem cells
I/R: ischemia-reperfusion
IFA/AAR: IFA per area at risk
IFA: infarct area
IHD: ischemic injury heart disease
KPC: K-ras; Trp53; Pdx-1-Cre (KPC)
LC3: Light chain 3
L-mimosine: L-mim
MPTP: 1-methyl-4-phenyl-1,2,3,6-tetrahydropyridine
MSCs: mesenchymal stem cells
NDD-CKD: Non-dialysis-dependent (NDD-)CKD
Nrf2: Erythroid 2-related factor 2
PD: parkinson’s diseases
PHDs: prolyl hydroxylase domain enzymes
RD: retinal detachment
ROP: retinopathy of prematurity
SOD2: Superoxide dismutase 2
TH: tyrosine hydroxylase
UUO: unilateral ureteral obstruction
VEGF: vascular endothelial growth factor
VHL: Von Hippel-Lindau
VLDL: very-low density lipoproteins
